# Dynamic vocal analysis: vocal functionality evaluation

**DOI:** 10.1590/2317-1782/20232021083en

**Published:** 2023-09-15

**Authors:** Mara Behlau, Glaucya Madazio, Rosiane Yamasaki

**Affiliations:** 1 Centro de Estudos da Voz - CEV - São Paulo, SP, Brasil.; 2 Universidade Federal de São Paulo - UNIFESP - São Paulo, SP, Brasil.

**Keywords:** Voice, Auditory perception, Speech acoustic, Spectrography, Dysphonia

## Abstract

Dynamic vocal analysis (DVA) is an auditory-perceptual and acoustic vocal assessment strategy that provides estimates on the biomechanics and aerodynamics of vocal production by performing frequency and intensity variation tasks and using voice acoustic spectrography. The objective of this experience report is to demonstrate the use of DVA in the assessment of vocal functionality of dysphonic and non-dysphonic individuals, with a special focus on the laryngeal musculature. Phonatory tasks consisted of sustained vowel, “a” or “é”, and/or connected speech, in three intensities (habitual, soft, and loud) and three frequencies (habitual, high, and low), as well as ascending and descending glissando. The adjustments of the laryngeal and paralaryngeal muscles can be inferred from the different DVA tasks. The main characteristics of the laryngeal muscles analyzed are control of glottic adduction, stretching, and shortening of the vocal folds; the main characteristics of the paralaryngeal musculature are mainly related to the vertical laryngeal position in the neck. While the sustained vowel evaluates the vocal functionality with a focus on the larynx, connected speech allows the evaluation of the articulatory adjustments employed. An acoustic spectrographic software can be used to visualize the performance of such tasks. The clinical application of the DVA will be exemplified using acoustic spectrography plates from normal and dysphonic voices, taken from a voice bank. Individuals who perform the DVA tasks in a balanced way, with adequate vocal quality and without phonatory effort, demonstrate good vocal functionality. On the other hand, difficulties in performing these tasks with worsening vocal quality and/or increased muscle tension may be indications of altered vocal functionality.

## INTRODUCTION

The assessment of the human voice consists of analyzing information from two dimensions: the patient and the clinician. The patient's perspective is examined by gathering information about the complaint and the impact of a voice problem, considering social, professional, daily life, and emotional expression aspects, along with specific evaluation aspects related to the quality of life and vocal performance impacts. This perspective can be obtained by using self-assessment instruments to measure the impact of dysphonia, once this information cannot be obtained through any other clinical procedure.

From the clinician's perspective, the voice assessment involves perceptual-auditory judgment of vocal quality, acoustic analysis of the vocal signal, physical examination, and larynx evaluation. Perceptual-auditory judgment of the voice quality is considered to be the gold standard in voice assessment since the voice is essentially a perceptual phenomenon^(1)^; however, this judgment is considered to be a subjective assessment, and its reliability is quite variable^(2)^, it depends on many factors related to speech material, the performed task, listening conditions and the judge experience^(3)^. On the other hand, the acoustic analysis is considered to be more objective and its main purpose is to record the voice to extract acoustic parameters or to perform descriptive analysis. The extraction of acoustic parameters provides the measurement of many vocal characteristics, some of which are more robust, such as the voice fundamental frequency (f_o_)^(4)^. The modern trend is to obtain combined measures^(5)^, such as cepstral measures^(6)^ and the Acoustic Vocal Quality Index (AVQi)^(7,8)^ as they have a high correlation with the perceptual judgment of the voice quality and do not consider only one isolated acoustic parameter. The descriptive analysis of the voice can be performed through the perceptual-visual judgment of the spectrogram, providing a qualitative assessment of the vocal sample. Some evaluation parameters have been proposed for this analysis^(9)^, they are highly dependent on the signal recording conditions^(10)^ and the evaluator's experience^(11)^. The most important aspects of descriptive analysis of the vocal signal are related to identifying the contribution of the source-filter, temporal aspects of the sound wave through visualization of the waveform, details of speech coarticulation, and individual characteristics.

Dynamic vocal analysis (DVA) is a simple and quick auditory-perceptual and acoustic vocal assessment strategy that provides estimates on the biomechanics and aerodynamics of vocal production by performing frequency and intensity variation tasks and using voice acoustic spectrography. Although previously described by Behlau and Pontes^(12)^, this new proposal involves an update, including a detailed procedure, objectives description, and additional tasks using voice acoustic spectrography. For the laryngeal functional assessment, three aspects must be considered: glottal adduction, vocal fold stretching, and vocal fold shortening. These movements are essential for voice production as they are responsible for controlling vocal loudness and pitch to produce soft, loud, high-pitched, and low-pitched sounds, which are essential variations for communication.

Glottal closure, along with subglottic pressure, is the main mechanism that controls vocal loudness. The greater the glottal closure and subglottic pressure, the higher the vocal loudness. On the other hand, the smaller the glottal closure and subglottic pressure, the lower the vocal loudness. The intrinsic muscles of the larynx responsible for glottal closure^(11,13-15)^ are the lateral cricoarytenoid (LCA), arytenoid (A), and thyroarytenoid (TA) muscles. In the DVA, the assessment of glottal adduction control is performed through emissions at habitual, soft, and loud loudness levels. Vocal fold tension is one of the main mechanisms to control vocal pitch^(13)^, where shortened vocal folds produce low-pitched sounds, while stretched vocal folds produce high-pitched sounds. The vocal fold stretching and shortening are carried out by the cricothyroid (CT) and thyroarytenoid (TA) muscles, respectively. In the DVA, the assessment of vocal fold flexibility is performed through emissions at a low and high pitch, as well as during glissando tasks.

The functionality of the extrinsic musculature can be assessed by analyzing the adjustments of the articulators in the vocal tract, especially during the production of high-pitched and low-pitched sounds. During vocal production, the extrinsic musculature of the larynx is responsible for maintaining the stability of the laryngeal skeleton so that the intrinsic musculature can act effectively^(14)^. In the production of high-pitched and low-pitched sounds, adjustments in both the intrinsic and extrinsic musculature of the larynx occurs simultaneously. The vertical movement of the larynx during tasks involving pitch variation is normal behavior.

In professional voice, trained singers tend to perform vocal pitch variation with a greater emphasis on the intrinsic musculature of the larynx, keeping the larynx flexible and stable in the neck. The DVA of singers provides data on the fine control of professional vocal production. One of the challenges for singers is to perform glottal adduction and vocal fold stretching and shortening movements in a nearly dissociated manner. Thus, these tasks allow assessing the singer's ability to vary vocal pitch while maintaining the same loudness, and, also, to vary loudness while controlling vocal pitch.

Based on the assumption that dysphonia can result in various losses and impairments of laryngeal functionality with different degrees of deviation, this article aims to demonstrate the use of DVA as an auditory-perceptual and acoustic assessment strategy for vocal functionality, with a focus on laryngeal musculature.

## DYNAMIC VOCAL ANALYSIS

The DVA can be performed with sustained vowels and connected speech.

A. **Sustained phonation**: assess aspects of the glottal source and it involves six tasks:

1. Self-reported habitual emission: sustained vowel “a” or “é” at habitual loudness and pitch. Baseline recording.
2. Self-selected soft emission: sustained vowel “a” or “é” at low loudness while maintaining phonation without whispering. Note: the ability to maintain slight adduction with consistent phonation and stability, and to maintain vocal pitch on the baseline recording, with the presence of at least 10 harmonics.
3. Self-selected loud emission: production of the vowel “a” or “é” at loud loudness without shouting. Note: vocal attack at the moment of adduction, strain, f_o_ compared to the baseline recording, emission quality such as the presence of subharmonics, involvement of vestibular folds, stability in the recording, and presence of at least 20 harmonics.
4. High-pitched emission: production of the vowel “a” or “é” at a high pitch. Note: the presence of excessive effort, maintenance of the baseline loudness recording, difficulty in performing or maintaining the emission, and characteristics of the emission quality such as vocal breaks.
5. Low-pitched emission: production of the vowel “a” or “é” at a low pitch. Note: vocal quality, maintenance of the loudness of the baseline recording, vocal stability, and phonatory comfort.
6. Glissando emission: sustained vowel “a” or “é” starting from a low pitch, ascending to a high pitch, and returning to the initial pitch. Note: pitch range in the task, symmetry in ascending and descending pitches, continuity in pitch transitions, and presence of vocal breaks.

B. **Connected speech:** it assesses adjustments of the articulators in the vocal tract, in terms of position and movement amplitude of the lips, jaw, tongue, and larynx. It also evaluates aspects of resonance and pneumophonic coordination, allowing an assessment of voice use in speech. Five tasks can be used:

1. Self-reported habitual emission: counting from 1 to 10 at habitual loudness and pitch. Baseline recording.
2. Self-selected soft emission: counting from 1 to 10 at low loudness while maintaining phonation without whispering. Note: the ability to maintain slight adduction of the vocal folds with control of soft loudness, resonance focus, articulation, and pneumophonic coordination.
3. Self-selected loud emission: counting from 1 to 10 at loud loudness, without shouting. Note: vocal attack at the moment of adduction, strain, involvement of vestibular folds, loud loudness control, resonance focus, articulation, and pneumophonic coordination.
4. High-pitched emission: counting from 1 to 10 at high pitch. Note: vocal quality, vertical laryngeal position, muscle tension, phonatory effort, pneumophonic coordination, speech rate, and characteristics of the emission quality such as vocal breaks.
5. Low-pitched emission: counting from 1 to 10 at low pitch. Note: vocal quality, vertical laryngeal position, muscle tension, phonatory effort, emission quality.

It is important to compare the emissions to the baseline recordings. The tasks should preferably be recorded using a voice acoustic spectrography program. The spectrographic analysis parameters should include a narrow-band spectrogram (40 Hz) at a sampling rate of 11 kHz for vowels and 22 kHz for continuous speech. Gray-scale spectrograms allow for more reliable analysis; however, color spectrograms provide better visual highlighting of the obtained changes.

The clinical application of DVA will be exemplified by spectrographic analysis plates of both normal and dysphonic individuals, taken from a voice database.

## PRESENTATION OF THE DVA SPECTROGRAPHIC ANALYSIS PLATES

**Figure 1:**
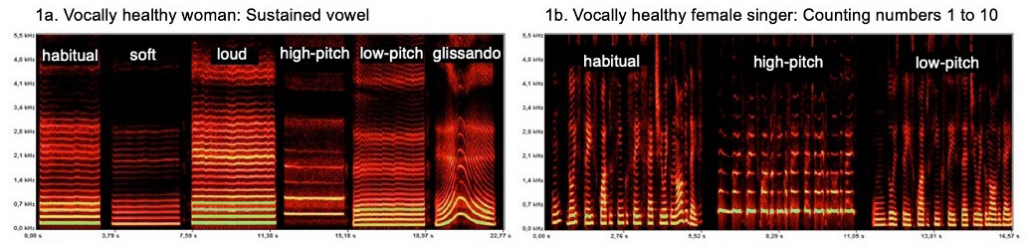
DVA from two vocally healthy women. 1a. DVA during sustained phonation of the vowel “é” in habitual, soft, and loud loudness and in high-pitch, low-pitch, and glissando; 1b. DVA from a female singer while counting numbers 1 to 10 in habitual, high, and low pitch emissions (Fono View 4.0 program)

**Figure 2:**
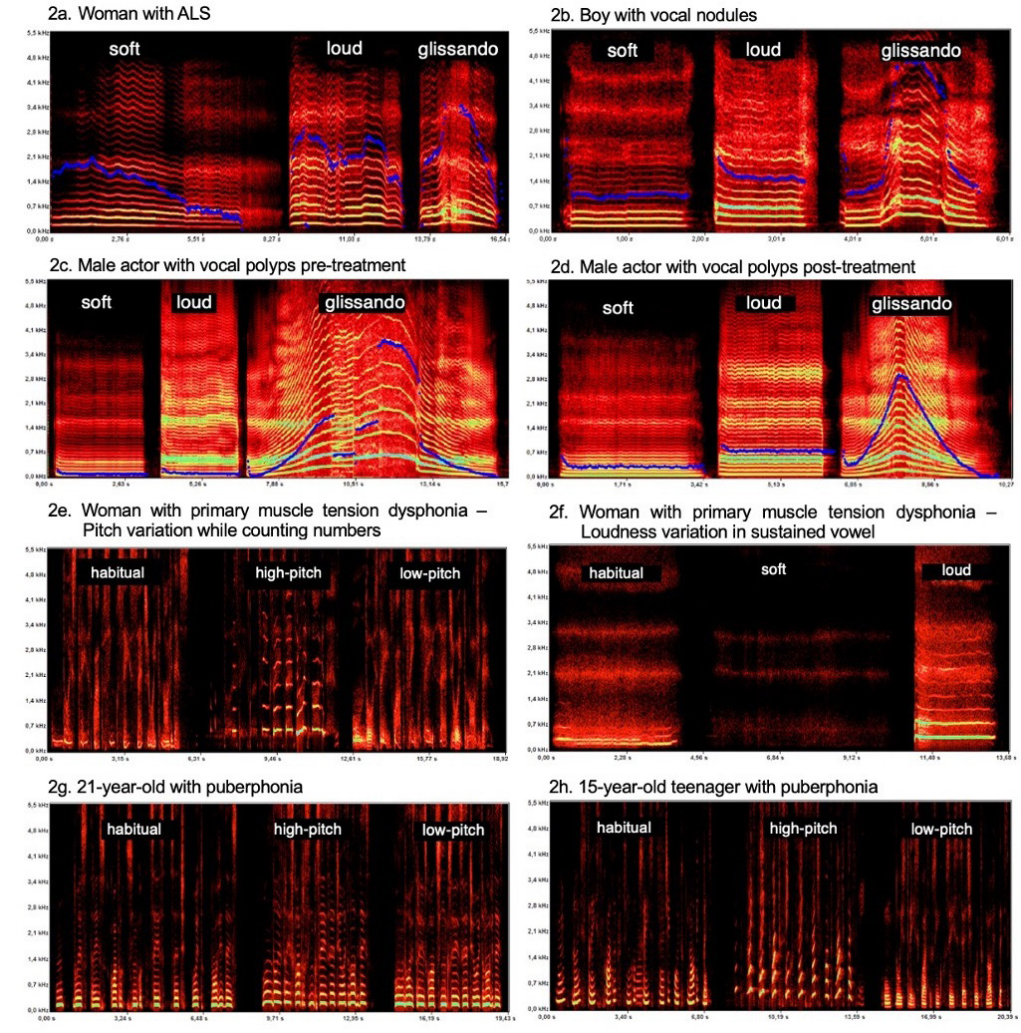
DVA from six dysphonic patients. 2a to 2d. DVA during sustained phonation of the vowel “é” in soft and loud loudness and in glissando. 2a) 53-year-old woman with ALS; 2b) 11-year-old boy with vocal nodules; 2c and 2d) Pre and post-treatment comparison of a 28-year-old male actor with vocal polyps; 2e and 2f. dubbing female artist with primary muscle tension dysphonia. 2e) DVA while counting from 1 to 10 at habitual, high-pitched, and low-pitched emissions; 2f) DVA during sustained phonation of the vowel “é” in habitual, soft, and loud loudness; 2g and 2h. Two male patients with puberphonia. DVA while counting numbers 1 to 10 in habitual, high, and low pitch emissions (FonoView 4.0 program).

To demonstrate the use of DVA in clinical practice, we selected 8 spectrographic plates from vocally healthy and dysphonic individuals while performing different phonatory tasks, Figures 1 and 2. The acoustic spectrography software used was FonoView 4.0, by CTS *Informática*.

Figure 1a shows the spectrogram of the DVA for a vocally healthy woman during sustained phonation of the vowel “é” with loudness and pitch variation. The stability of the trace is evident in all tasks, the soft and loud spectrographic trace indicate changes in glottic adjustments while maintaining stable f_o_; there is a tendency for higher f_o_ values associated with loud loudness. On the other hand, the soft loudness indicates maintenance and stability of the trace, which is rarely achieved by dysphonic patients. There are clear changes in f_o_ for high and low-pitched emissions. The high-pitched emission shows a slight alteration in the vocal quality, with increased noise between the harmonics and decreased intensity of the higher harmonics, which is consistent with the physiological adjustment of higher frequencies.

Figure 1b shows the DVA of a vocally healthy female singer while counting numbers 1 to 10 in habitual, high, and low pitch emissions. The spectrographic trace in the three emissions presents good definition, precise articulation, and high energy in the spectrum, consistent with adequate vocal projection. The frequency variation is adequate, indicating good vocal flexibility. The harmonics are more widely spaced in the high-pitched emission and closer together in the low-pitched emission. In comparison with the habitual pitch, there was a slight decrease in loudness in the low-pitched emission.

Figure 2 presents the DVA for six dysphonic patients. Figure 2a. is a 53-year-old woman with Amyotrophic Lateral Sclerosis - ALS. There is a clear instability to maintain the f_o_ trace, both in soft and loud emissions; there is also a change in the vocal quality with a decrease in frequency in soft emissions, reaching the basal register, in addition to variability in the number of harmonics. There is a wide fluctuation in maintaining the f_o_, with frequency breaks in loud emissions and bifurcation of the fundamental at the beginning of this task. In the glissando, fundamental bifurcation and probable involvement of vestibular folds in the high-pitched region of the trace are also observed. The DVA findings reflect the neurological alterations associated with ALS, characterized by mixed dysarthria (hypokinetic and hyperkinetic).

Figure 2b is from an 11-year-old boy with vocal nodules. There is a vocal attack with vocal breaks in soft phonation and sudden onset in loud phonation. In the soft emission, there is a spectral decrease, while in the loud emission, there is a higher f_o_ when compared to the soft emission, with strain, roughness, and vocal breaks at the end of the emission. The glissando is irregular, with excessive strain, frequency jumps, and bifurcation of the f_o_ in the high-pitched region of the task.

Figures 2c and 2d compare two traces, pre, and post-treatment of vocal polyps of a 28-year-old male actor. The pre-treatment DVA shows abrupt vocal attacks and strain in all three tasks; soft emission with a high number of harmonics due to strain; glissando with frequency breaks in both ascending and descending scales and, the presence of subharmonics probably due to the polyp mass, resulting in noisy trace between the harmonics. In the post-treatment moment, there is a more regular emission in all three tasks: f_o_ is less low-pitched in both soft and loud emissions; isochronic vocal attack in soft emission (*ataque vocal isocronico* in Brazilian Portuguese, it is characterized by a synchronized and coordinated start of vocal fold vibration during speech production); greater regularity in the harmonics; no vocal breaks in the glissando and less strain, although with reduced frequency-range in the high-pitched region.

Figures 2e and 2f illustrate the DVA of a dubbing female artist with primary muscle tension dysphonia - pMTD. Figure 2e displays the counting from 1 to 10 at habitual, high-pitched, and low-pitched emissions. At the habitual pitch, a high vocal frequency is observed, a low number of harmonics, frequency bifurcation, and the presence of subharmonics. In the high-pitched emission, there are strain, vocal breaks, and ascending frequency breaks. In the low-pitched emission, the vowels have few harmonics, noise in the low frequencies, the substitution of harmonics with noise, and vocal breaks. Figure 2f presents the sustained vowel “é” at habitual, soft, and loud loudness. In habitual loudness, there is a limited number of harmonics, frequency bifurcation, and the presence of noise. In the soft loudness, there is no phonation. In the loud loudness, there is a better definition of the first harmonics and the presence of noise in the trace. This patient’s DVA shows significant impairment in vocal functionality, both in pitch control and loudness control.

Figures 2g and 2h present the DVA of two patients with puberphonia while counting from 1 to 10 at habitual, high-pitched, and low-pitched emission. Figure 2g is from a 21-year-old patient who has minimal pitch variation in all three tasks. In the perceptual-auditory judgment, a slight pitch variation is heard, and very similar vocal qualities; which indicates the use of the same laryngeal mechanism in all three emissions, with the predominance of the cricothyroid muscle (CT). Figure 2h is from a 15-year-old patient. Significant frequency variation is observed among the three emissions. In the habitual emission, the frequency is high-pitched, and the loudness is soft. In the high-pitched emission, the spectrogram displays upward harmonic markings, which are visual representations of the harmonics showing an ascending pattern of frequency progression. In perceptual-auditory judgment, there is no strain. In the low-pitched emission, the patient was able to switch laryngeal mechanisms, with significant activation of the thyroarytenoid muscle (TA). In perceptual-auditory judgment, the voice became considerably low-pitched and slightly rough. The comparison of both DVAs demonstrates that the vocal flexibility in these two cases of puberphonia is significantly different. The 15-year-old patient has greater flexibility in the laryngeal musculature compared to the 21-year-old patient.

The DVA analysis can be considered a useful clinical tool that provides intuitive interpretation directly related to vocal functionality. The spectrographic analysis of the DVA can be performed in any software with spectrographic analysis, including free options available on the internet.

## DISCUSSION

The ability to vary the dynamic range, with the emission of soft and loud sounds, and the phonatory range, with high-pitched and low-pitched emissions, is essential to express our emotions, generating richness in vocal quality, and reflecting the plasticity of the laryngeal mechanism. The greater the dynamic and phonatory range, the greater the vocal flexibility, which reflects vocal health and allows for adequate vocal expressiveness. In professional voice use, this flexibility contributes to the necessary quality and endurance that the professional activity requires.

The DVA analysis of a patient with vocal complaints or a person seeking vocal improvement allows the vocal functionality diagnosis and enables a comparative analysis of the outcomes from vocal training or rehabilitation.

The DVA combined with the acoustic spectrography favors the clinical reasoning regarding biomechanical and aerodynamic characteristics of vocal production, efficiently demonstrating the patients’ vocal functionality. Due to its simplicity and fast execution, the DVA can be used for both vocal assessment and monitoring during voice therapy. The conclusion of the DVA evaluation should include information about the auditory-perceptual and acoustic characteristics of the voice observed during phonatory tasks, such as changes in vocal quality and spectrographic trace.

## FINAL COMMENTS

The present article aimed to demonstrate the DVA clinical application, an auditory-perceptual and acoustic vocal assessment strategy that assesses vocal and laryngeal functionality, whether for vocal improvement or to analyze dysphonic patients’ vocal conditions and to follow-up treatment outcomes. This is an easy-to-implement strategy and it can be recorded using any voice acoustic spectrography software.
