# Supplementary material for: Dynamic vocal analysis: vocal functionality evaluation
Source: Codas. 2023 Sep 15;35(5):e20210083. doi: 10.1590/2317-1782/20232021083en (PMC10546986; doi:10.1590/2317-1782/20232021083en)
Supplement: Translation

# Translation: Campo dinâmico vocal: avaliação da funcionalidade da voz

**DOI:** 10.1590/2317-1782/20232021083pt

**Authors:** Mara Behlau, Glaucya Madazio, Rosiane Yamasaki

**Affiliations:** 1 Centro de Estudos da Voz - CEV - São Paulo, SP, Brasil.; 2 Universidade Federal de São Paulo - UNIFESP - São Paulo, SP, Brasil.

**Keywords:** Voz, Percepção auditiva, Acústica da Fala, Espectrografia, Disfonia

## Abstract

O campo dinâmico vocal (CDV) é uma estratégia de avaliação vocal perceptivo-auditiva e acústica que oferece estimativas sobre a biomecânica e a aerodinâmica da produção vocal por meio da realização de tarefas de variação de frequência e de intensidade e do uso da espectrografia acústica da voz. Esse relato de experiência tem como objetivo demonstrar a aplicação do CDV na avaliação da funcionalidade vocal de indivíduos disfônicos e não-disfônicos, com foco especial na musculatura laríngea. As tarefas fonatórias envolvem emissão de vogal sustentada e/ou de fala encadeada em três intensidades autosselecionadas (habitual autorreferida, fraca e forte) e em três frequências (habitual autorreferida, aguda, grave), além do glissando. As tarefas de variação de intensidade e de frequência possibilitam a avaliação da musculatura laríngea no controle da adução glótica e do alongamento e encurtamento das pregas vocais. A tarefa de variação de frequência permite também a análise da musculatura extrínseca no controle da posição vertical da laringe no pescoço. Enquanto a vogal sustentada avalia a funcionalidade vocal com foco na laringe, a fala encadeada permite a avaliação dos ajustes articulatórios empregados. A aplicação do CDV será demonstrada por meio de pranchas espectrográficas de indivíduos normais e disfônicos. Indivíduos vocalmente saudáveis realizam as tarefas do CDV de forma equilibrada, com qualidade vocal adequada e sem esforço fonatório, denotando boa funcionalidade vocal. Por outro lado, indivíduos com dificuldade na realização das tarefas do CDV, com piora da qualidade vocal e/ou com aumento da tensão muscular, podem apresentar funcionalidade vocal alterada.

## INTRODUÇÃO

A avaliação da voz humana compõe-se da análise de informações em duas dimensões: a do paciente e a do clínico. A perspectiva do paciente é analisada pela coleta de informações sobre a queixa e o impacto de um problema de voz, considerando-se aspectos sociais, profissionais, da vida diária e da expressão das emoções, além da avaliação específica de aspectos da qualidade de vida e dos impactos no desempenho vocal. Essa perspectiva pode ser obtida com a utilização de instrumentos de autoavaliação do impacto da disfonia, sendo que tais informações não podem ser obtidas por nenhum outro procedimento clínico.

Sob a perspectiva do clínico, a avaliação da voz envolve a análise perceptivo-auditiva da qualidade vocal, a análise acústica do sinal sonoro, o exame físico e a avaliação otorrinolaringológica da laringe. A análise perceptivo-auditiva é o padrão ouro da avaliação vocal pelo fato da voz ser essencialmente um fenômeno perceptivo^(1)^; contudo, é considerada uma avaliação subjetiva e a confiabilidade desse procedimento pode ser bastante variável^(2)^, sendo dependente de inúmeros fatores relacionados ao material de fala, tarefa executada, condições da escuta e experiência do avaliador^(3)^. A análise acústica, por sua vez, é considerada mais objetiva e tem como principal função o registro da voz para extração de parâmetros acústicos ou para análise descritiva. A extração de parâmetros acústicos oferece a mensuração de diversas características vocais, algumas delas mais robustas, como a medida da frequência fundamental (f_o_) da voz^(4)^. A tendência moderna é a obtenção de medidas combinadas^(5)^, como as medidas cepstrais^(6)^ e o Acoustic Vocal Quality Index (AVQi)^(7,8)^ por terem uma correlação mais elevada com a análise perceptivo-auditiva, e não apenas considerar parâmetros isolados. A análise descritiva da voz pode ser feita pela avaliação perceptivo-visual do traçado espectrográfico e oferece uma avaliação qualitativa da amostra vocal. Alguns parâmetros de avaliação foram propostos para essa análise^(9)^, sendo altamente dependentes das condições de registro do sinal^(10)^ e da experiência do avaliador^(11)^. Os aspectos mais importantes da análise descritiva do sinal vocal estão relacionados com a identificação da contribuição da fonte-filtro, dos aspectos temporais da onda sonora com visualização do traçado, dos detalhes da coarticulação da fala e das características individuais do sujeito.

O campo dinâmico vocal (CDV) é uma estratégia de avaliação vocal perceptivo-auditiva e acústica simples e de rápida execução que oferece estimativas sobre a biomecânica e a aerodinâmica da produção vocal a partir da realização de tarefas de variação de frequência e de intensidade e do uso da espectrografia acústica da voz. Embora tenha sido anteriormente descrito por Behlau e Pontes^(12)^, essa nova proposta envolve uma atualização, incluindo o detalhamento do procedimento, a descrição dos objetivos e o acréscimo da realização das tarefas com a utilização da espectrografia acústica da voz. Para a avaliação da funcionalidade laríngea, três aspectos devem ser considerados: adução glótica, alongamento e encurtamento das pregas vocais. Esses movimentos são fundamentais para a produção da voz, pois respondem pelos controles de intensidade e de frequência vocal, por meio das emissões de fracos, fortes, agudos e graves, variações essenciais para a comunicação.

O fechamento glótico, juntamente com a pressão subglótica, são os principais mecanismos de controle da intensidade vocal. Quanto maiores forem o fechamento glótico e a pressão subglótica, maior a intensidade vocal. Por outro lado, quanto menores forem o fechamento glótico e a pressão subglótica, menor a intensidade vocal. Os músculos intrínsecos da laringe responsáveis pelo fechamento glótico^(11,13-15)^ são o cricoaritenóideo lateral-CAL, os aritenóideos-A e o tireoaritenóideo-TA. No CDV, a avaliação do controle de adução glótica é realizada por meio de emissões em intensidade habitual, fraca e forte. A tensão das pregas vocais é um dos principais mecanismos de controle de frequência da voz^(13)^, sendo que pregas encurtadas produzem sons graves e pregas vocais alongadas produzem sons agudos. Os movimentos de alongamento e de encurtamento das pregas vocais são realizados pelos músculos cricotireóideo-CT e tireoaritenóideo-TA, respectivamente. No CDV, a avaliação da flexibilidade das pregas vocais é realizada por meio de emissões em frequência grave e aguda e durante o glissando.

A funcionalidade da musculatura extrínseca pode ser verificada pela análise dos ajustes dos articuladores do trato vocal, principalmente na emissão de agudos e de graves. Durante a produção vocal, a musculatura extrínseca da laringe tem a função de manter a estabilidade do esqueleto laríngeo para que a musculatura intrínseca atue de forma efetiva^(14)^. Na emissão de agudos e de graves, os ajustes da musculatura intrínseca e extrínseca da laringe ocorrem concomitantemente. A movimentação vertical da laringe durante tarefas de variação de frequência é um comportamento normal.

Na voz profissional, cantores treinados tendem a realizar a variação de frequência vocal com ênfase maior na musculatura intrínseca da laringe, mantendo a laringe flexível e estável no pescoço. O CDV de cantores oferece dados sobre o controle fino da emissão profissional. Um dos desafios do cantor é realizar os movimentos de adução glótica e de alongamento/encurtamento de pregas vocais de forma praticamente dissociadas. Assim, as tarefas possibilitam verificar a habilidade do cantor em variar a frequência vocal mantendo-se a mesma intensidade, e, por outro lado, variar a intensidade com controle da frequência vocal.

Partindo do pressuposto de que uma disfonia pode implicar em perdas diversas e em variados graus da funcionalidade laríngea, o presente artigo tem como objetivo demonstrar o uso do CDV como uma estratégia de avaliação perceptivo-auditiva e acústica descritiva da funcionalidade vocal, com foco na musculatura laríngea.

## CAMPO DINÂMICO VOCAL

O CDV pode ser realizado com tarefa de vogal sustentada e de fala encadeada.

A. **Emissão sustentada**: avalia aspectos da fonte glótica e envolve seis tarefas:

1. Emissão habitual autorreferida: vogal “a” ou “é” em intensidade e frequência habituais. Registro de base.
2. Emissão fraca autosselecionada: vogal “a” ou “é” em intensidade fraca, mas mantendo a sonoridade, sem sussurrar. Observar: habilidade de manter adução leve com sonoridade constante e estabilidade, de manter a frequência vocal do registro de base e de apresentar pelo menos 10 harmônicos.
3. Emissão forte autosselecionada: produção da vogal “a” ou “é” em intensidade forte, mas sem gritar. Observar: ataque vocal no momento da adução, tensão, f_o_ em relação ao registro de base, qualidade da emissão como a presença de sub-harmônicos, envolvimento de pregas vestibulares, estabilidade no registro e pelo menos 20 harmônicos.
4. Emissão aguda: produção da vogal “a” ou “é” em frequência aguda. Observar: presença de esforço excessivo, manutenção da intensidade do registro de base, dificuldade para realizar a tarefa e/ou manter a emissão, qualidade da emissão como quebras de sonoridade e de frequência.
5. Emissão grave: produção da vogal “a” ou “é” em frequência grave. Observar: qualidade vocal, manutenção da intensidade do registro de base, estabilidade vocal e conforto fonatório.
6. Emissão em glissando: vogal “a” ou “é”, começando por um tom grave, subindo ao agudo e retornando ao tom inicial. Observar: extensão de frequências na tarefa, simetria na subida e na descida dos tons, continuidade no deslocamento das frequências e presença de quebras de frequência ou de sonoridade.

B. **Emissão de fala encadeada:** avalia os ajustes dos articuladores do trato vocal, tanto em relação à posição como em relação à amplitude de movimento dos lábios, da mandíbula, da língua e da própria laringe. Além disso, avalia também aspectos de ressonância e de coordenação pneumofônica, permitindo a apreciação do uso da voz na fala. Cinco tarefas podem ser utilizadas:

1. Emissão habitual autorreferida: contagem de 1 a 10 em intensidade e frequência habituais. Registro de base.
2. Emissão fraca autosselecionada: contagem de 1 a 10 em fraca intensidade, mantendo a sonoridade, sem sussurrar. Observar: habilidade de manter adução leve das pregas vocais com controle da intensidade fraca, foco de ressonância, articulação e coordenação pneumofônica.
3. Emissão forte autosselecionada: contagem de 1 a 10 em forte intensidade, sem gritar. Observar: presença de ataque brusco, tensão excessiva, envolvimento de pregas vestibulares, controle da intensidade forte, foco de ressonância, articulação e coordenação pneumofônica.
4. Emissão aguda: contagem de 1 a 10 em frequência aguda. Observar: qualidade vocal, PVL, tensão muscular, esforço fonatório, coordenação pneumofônica, velocidade de fala, qualidade da emissão como quebras de sonoridade ou de frequência.
5. Emissão grave: contagem de 1 a 10 em frequência grave. Observar: qualidade vocal, PVL, tensão muscular, esforço fonatório, qualidade da emissão.

Importante comparar as emissões aos registros de base. O registro das tarefas deve ser feito, preferencialmente, com a utilização de um programa de espectrografia acústica da voz. Os parâmetros da análise espectrográfica devem incluir: espectrograma de faixa estreita (40 Hz), taxa de amostragem de 11 kHz para as tarefas com as vogais e 22 kHz para as tarefas com fala encadeada. Os espectrogramas em gradação de cinza permitem uma análise mais confiável; contudo, os espectrogramas coloridos destacam mais as mudanças obtidas.

A aplicação clínica do CDV será exemplificada por meio de pranchas de espectrografia acústica da voz de indivíduos normais e disfônicos, retiradas de um banco de vozes.

## APRESENTAÇÃO DOS REGISTROS ESPECTROGRÁFICOS DO CDV

Para demonstrarmos o uso do CDV na prática clínica, selecionamos oito exemplos de registros espectrográficos de indivíduos vocalmente saudáveis e disfônicos durante a realização das diferentes tarefas fonatórias, Figuras 1 e 2. O programa de espectrografia acústica da voz utilizado foi o FonoView 4.0, CTS Informática.

**Figura 1:** CDV de duas mulheres jovens vocalmente saudáveis. 1a. CDV com tarefas de VOGAL “é” em intensidade habitual, fraca e forte e em frequência aguda, grave e em glissando; 1b. CDV de uma cantora com tarefas de contagem de 1 a 10 em frequência habitual, aguda e grave (programa Fono View 4.0)

**Figura 2:** CDV de seis pacientes disfônicos. 2a a 2d. CDV com tarefas de VOGAL “é” em intensidade fraca e forte e em glissando. 2a) Mulher de 53 anos com ELA; 2b) Menino de 11 anos com nódulos vocais; 2c e 2d) Comparação pré e pós-tratamento de pólipo de prega vocal de um ator de 28 anos por microcirurgia laríngea e reabilitação vocal; 2e e 2f. Dubladora de 53 anos com disfonia por tensão muscular. 2e) CDV com tarefa de contagem de números de 1 a 10 em frequência habitual, aguda e grave; 2f) CDV com tarefa de vogal “é” em intensidade habitual, fraca e forte; 2g e 2 h. Pacientes do sexo masculino com muda vocal incompleta. CDV com tarefas de contagem de números de 1 a 10 em frequência habitual, aguda e grave (programa FonoView 4.0)

A Figura 1a mostra a espectrografia do CDV de uma mulher vocalmente saudável durante a emissão da vogal sustentada “é” com variação de intensidade e de frequência. A estabilidade do traçado em todas as tarefas é visível; o traçado espectrográfico do fraco e do forte indica mudança de ajustes glóticos, com a f_o_ praticamente estável; observa-se uma tendência da associação do forte com elevação da f_o_. Por outro lado, a fraca intensidade indicou a manutenção e estabilidade do traçado, o que dificilmente é atingido por pacientes disfônicos. Nota-se também nítida mudança da f_o_ nas emissões aguda e grave. A emissão aguda mostra uma leve mudança da qualidade vocal, com maior ruído entre os harmônicos e menor intensidade dos harmônicos mais agudos, compatível com o ajuste fisiológico de frequências agudas.

A Figura 1b mostra o CDV de uma cantora vocalmente saudável durante a contagem de 1 a 10 em frequência habitual, aguda e grave. Nota-se boa definição do traçado espectrográfico nas três emissões, articulação precisa e grande energia no espectro, demonstrando adequada projeção vocal. Pode-se observar adequada variação de frequência, o que denota boa flexibilidade vocal. Os harmônicos são mais espaçados no agudo e mais próximos no grave. Em relação à frequência habitual, houve leve diminuição da intensidade vocal no grave.

A Figura 2 mostra o CDV de seis pacientes disfônicos. A Figura 2a. é de uma mulher de 53 anos com Esclerose Lateral Amiotrófica - ELA. Observa-se nítida instabilidade na manutenção do traçado da f_o_, tanto na emissão de sons fracos como nos fortes; Observa-se também mudança de qualidade vocal, com queda de frequência nos fracos chegando a atingir o registro basal, além de variabilidade no número dos harmônicos nesse trecho; há flutuação muito ampla na manutenção da f_o_, com quebras de frequências na emissão dos fortes e bifurcação da fundamental no início dessa tarefa; no glissando, também se observa bifurcação da fundamental e provável envolvimento de pregas vestibulares na região aguda do traçado; os achados do CDV dinâmico refletem as alterações neurológicas da ELA, caracterizada por uma disartria mista, hipocinética e hipercinética.

A Figura 2b é de um menino de 11 anos de idade com nódulos vocais. Nota-se a presença de ataque vocal com quebra de sonoridade na emissão dos fracos e brusco nos fortes; na emissão dos sons fracos identifica-se queda espectral rápida, enquanto que na emissão dos sons fortes observa-se f_o_ mais aguda do que nos fracos, com tensão, rugosidade e quebra de sonoridade ao final da emissão; o glissando é irregular, com tensão excessiva, saltos de frequência e bifurcação da f_o_ na região aguda da tarefa.

As Figuras 2c e 2d mostram a comparação de dois traçados, pré e pós-tratamento de pólipo de prega vocal, de um ator de 28 anos. O CDV pré-tratamento mostra ataques vocais bruscos e tensão nas três tarefas, emissão dos fracos com grande número de harmônicos pela tensão, glissando com quebras de frequência na escala ascendente e descendente, presença de sub-harmônico provavelmente produzido pela massa do pólipo, com traçado de ruído entre os harmônicos; no pós-tratamento observa-se emissão mais regular nas três tarefas, com f_o_ menos grave nas emissões fraca e forte, ataque isocrônico na emissão dos fracos, maior regularidade nos harmônicos, glissando sem quebras e com menor esforço, embora com menor extensão de frequências nos agudos.

As Figuras 2e e 2f mostram o CDV de uma dubladora com disfonia por tensão muscular primária-DTMp. A Figura 2e mostra a contagem de 1 a 10 em frequência habitual, aguda e grave. Em frequência habitual, observa-se frequência vocal elevada, número de harmônicos bastante reduzido, presença de bifurcação de frequência e de sub-harmônicos. No agudo, observa-se tensão, quebras de sonoridade e quebra de frequência ascendente. No grave, nota-se também vogais com poucos harmônicos, ruído nas frequências graves, substituição de harmônicos por ruído e quebras de sonoridade. A Figura 2f mostra a vogal sustentada “é” em intensidades habitual, fraca e forte. Na emissão habitual, observa-se número restrito de harmônicos, bifurcação de frequência e presença de ruído. Em intensidade fraca, ocorre ausência de sonoridade. Em intensidade forte observa-se melhor definição dos primeiros harmônicos e presença de ruído no traçado. A espectrografia do CDV dessa paciente demonstra importante comprometimento da funcionalidade vocal tanto no controle de frequência como no de intensidade.

As Figuras 2g e 2h mostram o CDV de dois pacientes com muda vocal incompleta durante contagem de 1 a 10 em frequências habitual, aguda e grave. A Figura 2g é de um paciente de 21 anos que apresenta pouca variação de frequência nas três tarefas. Auditivamente, escuta-se pequena variação de frequência e qualidades vocais muito semelhantes, o que demonstra o provável uso do mesmo mecanismo laríngeo nas três emissões, com predomínio importante do músculo cricotireóideo -CT. A Figura 2h é de um paciente de 15 anos de idade. Observa-se grande variação de frequência entre as três emissões. Na emissão habitual, a frequência é aguda e a intensidade fraca. Na emissão aguda, a espectrografia mostra marcação de harmônicos com direção ascendente. Auditivamente, não se escuta tensão. Na frequência grave, o paciente conseguiu realizar a mudança do mecanismo laríngeo, com importante ativação do músculo tireoaritenóideo -TA. Auditivamente, a voz tornou-se grave e levemente rugosa. A comparação dos dois CDVs mostra que a flexibilidade vocal desses dois casos de muda vocal incompleta é muito diferente, sendo que o paciente de 15 anos apresenta maior flexibilidade da musculatura laríngea.

A análise do CDV pode ser considerada uma ferramenta clínica útil, de interpretação intuitiva e diretamente relacionada à funcionalidade vocal. A análise espectrográfica do CDV pode ser analisada em qualquer programa de análise espectrográfica, incluindo os gratuitos disponíveis na internet.

## DISCUSSÃO

A possibilidade de variar a extensão dinâmica, com emissão de fraco e forte, e a extensão fonatória, com a emissão agudo e grave, é essencial para a expressão de nossas emoções, gerando riqueza na qualidade vocal e refletindo a plasticidade do mecanismo laríngeo. Quanto maior a extensão dinâmica e fonatória, maior a flexibilidade vocal, o que reflete saúde vocal e que permite adequada expressividade vocal. Quando se trata do uso profissional da voz, essa flexibilidade colabora para a qualidade e a resistência necessárias para a profissão.

A análise do CDV de um paciente com queixa de voz ou de um indivíduo que busca o aperfeiçoamento vocal possibilita o diagnóstico da funcionalidade vocal e também a análise comparativa do resultado de um programa de desenvolvimento vocal ou de reabilitação.

O CDV associado à espectrografia acústica da voz favorece o direcionamento do raciocínio clínico quanto às características biomecânicas e aerodinâmicas da produção vocal, mostrando de forma eficiente a condição da funcionalidade vocal do paciente. Por ser uma tarefa simples e de rápida execução, o CDV pode ser utilizado tanto na avaliação vocal como durante a terapia de voz para fins de monitoramento. A conclusão da avaliação do CDV deve conter informações sobre as características perceptivo-auditivas e acústicas da voz observadas durante a realização das tarefas fonatórias, como mudanças da qualidade vocal e do traçado espectrográfico.

## COMENTÁRIOS FINAIS

O presente artigo teve como objetivo demonstrar a aplicação clínica do CDV, uma estratégia de avaliação perceptivo-auditiva e acústica descritiva que avalia a funcionalidade laríngea vocal, seja para fins de aperfeiçoamento vocal ou para analisar as condições vocais de um paciente com distúrbio vocal e acompanhar os resultados de um tratamento. A estratégia é de fácil execução e pode ser registrada em qualquer programa de espectrografia acústica da voz.
